# Translational Considerations to Improve Response and Overcome Therapy Resistance in Immunotherapy for Hepatocellular Carcinoma

**DOI:** 10.3390/cancers12092495

**Published:** 2020-09-03

**Authors:** Sophia Heinrich, Darko Castven, Peter R. Galle, Jens U. Marquardt

**Affiliations:** 1 Laboratory of Human Carcinogenesis, Liver Carcinogenesis Section, Center for Cancer Research, National Cancer Institute, National Institutes of Health, Bethesda, MD 20892, USA; sophia.franck@nih.gov; 2 Department of Medicine I, Lichtenberg Research Group for Molecular Hepatocarcinogenesis, University Medical Center, 55131 Mainz, Germany; darko.castven@uksh.de; 3 Lichtenberg Research Group for Molecular Hepatocarcinogenesis, Department of Medicine I, University Medical Center Schleswig Holstein, 23538 Luebeck, Germany; 4 Department of Medicine I, University Medical Center, 55131 Mainz, Germany

**Keywords:** hepatocellular carcinoma, immunotherapy, translational approaches, combination therapies, therapy resistance

## Abstract

**Simple Summary:**

Immunotherapeutic approaches became a promising treatment option and an intensive field of research in liver cancer. Despite promising results in preclinical studies, only moderate response rates have been reported in phase III clinical trials and predictive biomarkers are still missing. Therefore, translational considerations are important to overcome resistance to immunotherapy. This article reviews potential predictors for response to immunotherapy in hepatocellular carcinoma (HCC) as well as potential mechanisms for therapy resistance. Further, we will discuss translational considerations to overcome therapy resistance in HCC and improve overall response rates.

**Abstract:**

Over the last decade, progress in systemic therapies significantly improved the outcome of primary liver cancer. More recently, precision oncological and immunotherapeutic approaches became the focus of intense scientific and clinical research. Herein, preclinical studies showed promising results with high response rates and improvement of overall survival. However, results of phase III clinical trials revealed that only a subfraction of hepatocellular carcinoma (HCC) patients respond to therapy and display only moderate objective response rates. Further, predictive molecular characteristics are largely missing. In consequence, suitable trial design has emerged as a crucial factor for the success of a novel compound. In addition, increasing knowledge from translational studies indicate the importance of targeting the tumor immune environment to overcome resistance to immunotherapy. Thus, combination of different immunotherapies with other treatment modalities including antibodies, tyrosine kinase inhibitors, or local therapies is highly promising. However, the mechanisms of failure to respond to immunotherapy in liver cancer are still not fully understood and the modulation of the immune system and cellular tumor composition is particularly relevant in this context. Altogether, it is increasingly clear that tailoring of immunotherapy and individualized approaches are required to improve efficacy and patient outcome in liver cancer. This review provides an overview of the current knowledge as well as translational considerations to overcome therapy resistance in immunotherapy of primary liver cancer.

## 1. Introduction 

Primary liver cancer, in particular hepatocellular carcinoma (HCC) ranks among the most common malignancies worldwide with a rising incidence in the Western world [1,2,3,4]. Between 80–90% of HCC cases develop in an inflammation-associated milieu [5], i.e., on the background of a pre-existing chronic liver disease and, most commonly, an advanced fibrosis or cirrhosis. Due to demographic changes in the distribution of diabetes mellitus type II and obesity, non-alcoholic fatty liver disease, or steatohepatitis (non-alcoholic fatty liver disease (NAFLD)/non-alcoholic steatohepatitis (NASH)) show a sharp increase in HCC numbers [6] and are considered as metabolic predispositions to liver cancer [7,8]. Numerous immune suppressor mechanisms that involve different immune cell types lead to immune evasion of the tumor and have been shown to contribute to HCC initiation and progression [9,10].

Despite well known risk factors, i.e., chronic viral hepatitis, alcohol consumption, and metabolic syndrome, the majority of HCC patients are diagnosed in late, non-resectable, and non-curative stages of the disease, when a considerable phenotypic and molecular heterogeneity renders HCC highly resistant to conventional chemotherapy and/or irradiation [11]. Until 2016, only limited systemic treatment options were available in advanced stages of HCC, namely sorafenib and regorafenib, tyrosine kinase inhibitors (TKI) [12,13,14]. Since then, only Lenvatinib (first-line), regorafenib, cabozantinib, all TKIs, and ramucirumab (second-line), a monoclonal antibody against VEGFR, have shown efficacy in phase III clinical trials [13,15,16,17]. Despite the approval of new and targeted therapy, patients’ prognosis remained limited to 12–13 months in first-line and 9–11 months in second-line therapy, and besides alpha-fetoprotein (AFP), there is no biomarker available for patient stratification [18].

Given the inflammatory background of HCC, the hepatic tumor microenvironment (TME) plays a pivotal role in tumor initiation, modulation of tumor invasiveness, metastatic spread as well as tumor suppression and immune surveillance of cancer cells [19]. Therefore, modern therapeutic approaches that focus on modulation of the TME are particularly promising. 

The liver is an immune tolerant organ due to its prominent role in protection against inappropriate immune responses. The inflammatory stimuli emerge as a consequence to exposition with major inflammatory processes mediated by a large antigenic load from the gastrointestinal tract trough blood from the portal vein [20]. In addition, the setting of a chronic liver inflammation or cirrhosis further reinforces the hepatic immune tolerance [21]. On a single cell level, it has been demonstrated that HCCs show a higher abundance of regulatory T cells (T_regs_) as well as their local clonal expansion within the tumor. Furthermore, a higher abundance of exhausted CD8 T cells is present in the tumor tissue [22]. This has a significant influence on tumor surveillance. Decreased number of tumor attacking immune cells such as T effector cells and more tumor supporting cells, e.g., MDSCs and T_regs_ lead to a disruption of the cellular composition during chronic liver diseases and is associated with patient outcome [23,24,25,26,27,28]. During hepatocarcinogenesis, several immunosuppressive effects have been detected that are associated with patient survival. Immune cell composition leading to anti-tumor immunity or tolerance is crucial for tumor growth or cell death. T_regs_ as well as myeloid derived suppressor cells (MDSC) accumulate in the liver and suppress antitumor immunity in HCC [9,29]. Macrophages, in the liver called Kupffer cells, suppress early HCC development; however, undergo a switch from M1 to M2 during tumor progression, which leads to a suppression of the adaptive immune system and support of the tumor [10,30,31,32,33]. Tumor associated macrophages (TAM) represent the predominant component of the innate immune system and promote tumor proliferation, angiogenesis and invasion [34,35] Furthermore, parenchymal cells such as endothelial cells, hepatic stellate cells (HSC), and hepatocytes influence effector functions of infiltrating lymphocytes [21]. This leads to an intratumoral loss of cytotoxic T cells, which is associated with tumor progression [21,35,36]. Natural killer (NK) cell, important players of innate immunity in the liver, show an impaired function in HCCs [29,37]. This dysfunctional and imbalanced immune system is a hallmark of cancer progression in HCC and is associated with patient prognosis. [38,39]

After the approval of immune checkpoint inhibitors (ICI) in melanoma and non-small cell lung cancer (NSCLC), immunotherapies have raised significant interest in other solid tumors including HCC. In 2017 and 2018, the FDA granted accelerated approval for the first immunotherapy agents, nivolumab and pembrolizumab or the combination of nivolumab and ipilimumab, for patients with advanced HCC after progression under sorafenib after promising results from phase II clinical trials [40,41,42]. Other checkpoint inhibitors are currently being investigated in clinical trials as single agents as well as in combination therapies [42,43,44,45]. A detailed list of currently approved immunotherapeutic agents can be found in Table 1. Nevertheless, immunotherapy in liver cancer has been challenging. Objective response rates are still low. Given the fact that only some patients respond to therapy, the various degrees of side effects such as autoimmune reactions need to be taken into account [40,46,47]. Thus, predictive biomarkers are urgently needed. Furthermore, there are no long-term data for those patients responding to therapy and even though there are some studies addressing a neoadjuvant treatment option, we do not have any strong data in curative settings yet. However, first results from combination therapies show a significant improvement in all clinical endpoints including overall survival and quality of life, which raises optimism for the future of this approach in primary liver cancer [48]. Even scenarios in adjuvant or neoadjuvant use are now under current discussion [49,50], but our overall understanding of the treatment response remains limited.

Given the success of immunotherapy in several tumor entities, we here review the potential predictors for response to immunotherapy in HCC. In addition, we are addressing potential mechanisms for therapy resistance. Finally, we discuss translational considerations to overcome therapy resistance in HCC.

## 2. General Strategies for Immunomodulatory Treatments in Primary Liver Cancer

**Figure 1:**
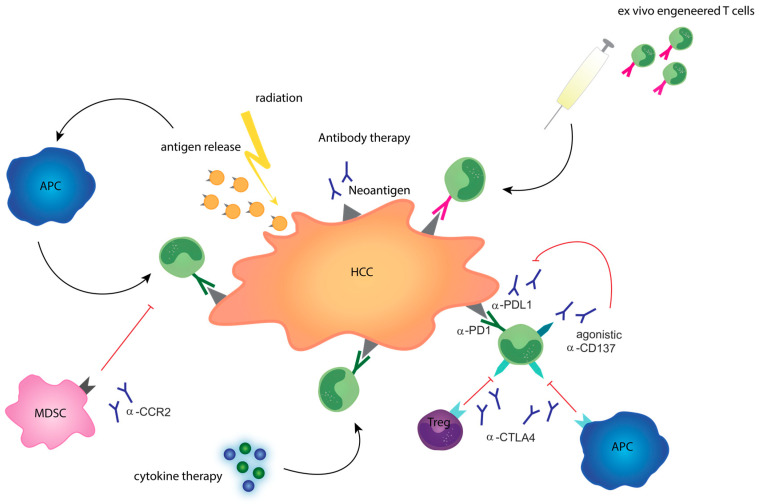
Overview of therapeutic approaches in immunotherapy. Targeted antibody therapy can block inhibitory signals, e.g., CTLA-4 or PD-1 and “unleash” anti-tumor immunity or block immunosuppressive mechanisms of the adaptive as well as the innate immune system. Besides a direct anti tumoral effect, irradiation leads to an antigen release that promotes antigen presentation by APCs and enhances anti-tumoral T cell response. Cytokine therapy is an option to enhance a general T cell response in the tumor. Ex-vivo engineered T cells or antibodies against tumor specific neoantigens induce a targeted anti-tumor response. Abbreviations: Myeloid derived suppressor cell (MDSC), antigen presenting cell (APC), regulatory T cell (Treg), cytotoxic T lymphocyte antigen 4 (CTLA4), programmed death protein (PD1), programmed death ligand 1 (PDL1).

There are different strategies to induce antitumor immune response that are currently under investigation in primary liver cancer involving both innate and adaptive immune systems. Specifically, targeting of checkpoint molecules as well as the interaction of T cells and antigen-presenting cells (APCs) have been of interest in recent years [51]. Neoantigens expressed on the tumor itself can also be used as targets for immunotherapy [52]. Local therapies and oncolytic viruses can promote neoantigen release even more, thereby further enhancing the antitumor immune response [53,54]. In addition, detailed information on tumor neoantigens can be explored to develop anti-tumor vaccines and autologous T cells can be manipulated and/or stimulated ex vivo before retransfer, e.g., chimeric antigen receptor (CAR) T cells or cytokine-induced killer cells (Figure 1) [55,56].

**Figure 2:**
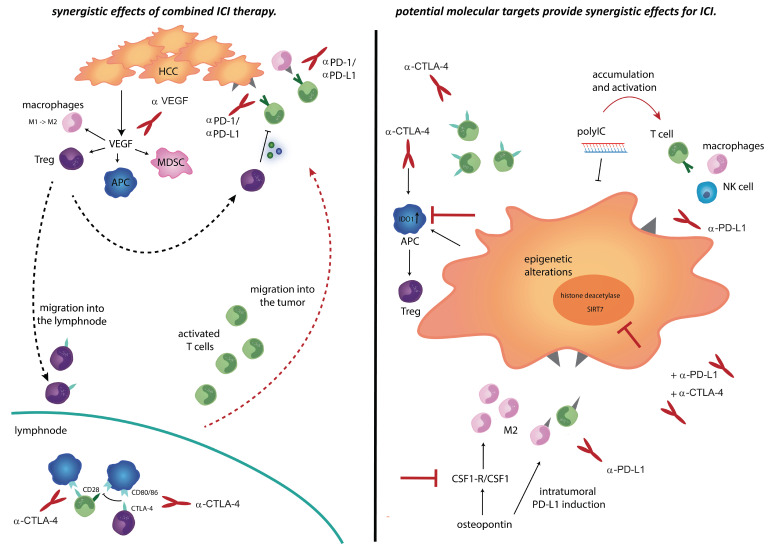
Translational consideration to overcome therapy resistance. Therapeutic approaches for sensitization to immunotherapy. **Left panel**: Anti-CTLA-4 or anti-angiogenic therapy increases recruitment and migration of activated T cells into the tumor. Anti-PD-1/anti PD-L1 therapy enhances cytolytic activity of T cells. **Right panel**: Anti-CTLA-4 treatment induced IDO1 expression in dendritic cells (DC). Indoleamine 2,3-dioxygenase 1 (IDO1) leads to activation of T_regs_ and causes resistance to anti-CTLA-4 therapy, while blocking of IDO could interrupt this mechanism of resistance. PolyIC inhibits tumor growth and leads to an accumulation and activation of immune cell subsets, whereas anti-PD-L1 therapy could provide synergistic effects. Osteopontin induces M2 migration into the tumor as well as PD-L1 induction. Targeted therapy of CSF1 in combination with anti-PD-L1 therapy might provide synergistic effects. Epigenetic regulations as synergistic effect for ICI therapy.

However, it is well known that immune escape and evasion of immune-mediated cytotoxicity are among the hallmarks of cancers and are often mediated by induction of an immunosuppressive microenvironment [57,58]. To overcome escape from immunosurveillance by cancer cells, therapeutic approaches focus on boosting antitumor response either by activation of cytotoxic immune cells or elimination of immune-suppressing cells. Furthermore, tumors also evade from the immune system by upregulation of programmed cell death ligand 1 (PD-L1) on cancer cells. Tumor immune cell interactions are based on two phases of T cell activation: an early priming phase in the lymph node and an effector phase in the tumor tissue. Involved in this process are APCs, that bind cancer antigens, migrate to the lymph node, and activate immature T cells. Activation of T cells in the priming phase can be blocked by upregulation of the checkpoint molecule cytotoxic T lymphocyte antigen 4 (CTLA-4) on T cells. CTLA-4 is also highly expressed on T_regs_ that inhibit antigen presentation on dendritic cells (DC). This is a cycle, that leads to less cytotoxic, more exhausted T cells and, thus, impaired anti-tumor response. Activation of T cells in the effector phase can be blocked by programmed death protein 1 (PD-1)/ programmed death ligand 1(PD-L1) that is expressed in tumor cell interaction. Both “breaks” can be effectively released by anti-PD-1, anti PD-L1, or anti CTLA-4 therapy and enhance anti-tumor immune response (Figure 2) [40,43,59,60].

In HCC, immunotherapy is an intensively studied field encompassing all the above mentioned antibody-based, cell-based, and vaccine-based treatment options [61]. In addition, the combination of different therapy regimes may provide a significant benefit (Figure 1) [43].

## 3. Predictors for Response or Resistance to Current Immune-Modulatory Therapies

Immunotherapy as a modern approach for cancer treatment has become a key topic in translational research over the last decade. After approval of the first PD-1/PD-L1 and CTLA-4 blocking antibodies for melanoma, checkpoint inhibitors are under intense investigation in many tumor entities. Unleashing of the immune system to attack the tumor seems to be an effective anti-tumor treatment. Many immunotherapies have been shown to be effective as monotherapies but also in combination with other immune-based and targeted approaches in preclinical and clinical studies [62,63,64,65,66]. However, despite good clinical efficacy in other tumor entities, response rates in HCC as well as cholangiocarcinoma are surprisingly low [40,67,68,69,70]. A common observation in HCC is the missing significance or lack of surrogate markers of response utilized in other entities. Thus, improved strategies to estimate therapy response would enable to stratify patients according to their clinical benefit and prevent unnecessary side effects caused by the therapy [40,70,71,72,73]. 

Mechanisms of resistance to immunotherapy are still not fully understood. Especially in the context of a possible pseudoprogression or even hyperprogression under immunotherapy, predictive biomarkers are urgently needed [74].

### 3.1. Tumor Characteristics and Tumor Infiltrating Lymphocytes as Predictors for Response

Multiple studies revealed potential molecular characteristics that are associated with immunotherapy response. However, up to now, no biomarker for HCC has been prospectively validated in authentic human patients. The most prominent biomarkers are PD-1 and PD-L1 expression on tumor tissue as well as on infiltrating immune cells.

Expression of PD-1/PD-L1 in HCC have been described in 17% (PD-L1) and 27% (PD-1) on immune and 10–20% (PD-L1) on tumor cells, using immunohistochemistry [40,41,75,76,77]. High PD-L1 expression in tumors itself is associated with more aggressive HCCs independent of immunotherapy [76,78].

Several translational studies investigated numbers of immune cells and respective activation of checkpoint molecules as possible biomarkers for immunotherapy response in HCC. In other entities such as NSCLC, PD-1^high^ T cells showed a higher capacity for tumor recognition, recruit other immune cells, and are predictive for response and overall survival under PD-1 therapy, which demonstrates that a distinct T cell subtype is needed for response to PD-1/PD-L1 therapy [79]. In HCC patients, high PD-1 expression in tumor tissue is connected to an exhausted immune cell phenotype with impaired effector function of tumor infiltrating lymphocytes (TIL), which contributes to immune evasion [75,80,81,82]. A recent study further demonstrated that PD-1, LAG3 (lymphocyte activation gene 3), TIM3 (T cell membrane protein 3), and CTLA-4 positive TILs are exhausted and functionally compromised, thus, induce lower levels of effector cytokines. Conversely, this phenotype could be reversed back to an effector phenotype with ICI [82]. 

Using sequencing and TCR analysis, another study investigated the distribution of mutational and neoantigen burden in different tumor regions as a possible driver for immune cell heterogeneity. Analysis of peptide binding affinity of these neoantigens revealed a correlation of the higher ones with TIL heterogeneity. However, the region with the highest TIL heterogeneity showed the lowest putatively immunogenic neoepitopes, suggesting that the adaptive immune response has edited the tumor to be less immunogenic [83].

Another study stratified HCC patients into CD8^+^PD-1^high^ and CD8^+^PD-1^low^. A gene signature that effectively predicted anti-PD-1 therapy response in several tumor entities was significantly enriched in corresponding PD-1^high^ expressers [75]. Furthermore, high frequencies of CD14^+^CD16^−^HLA-DR^high^monocytes was shown to predict therapy response in melanoma patients [84]. This phenotype was also elevated in PD-1^high^ expressers [75]. Both findings might provide an indirect surrogate of therapy response in PD-1^high^ HCC patients. Consistently, the PD-1^high^ HCCs also expressed markers such as LAG3 and TIM3 confirming the exhausted phenotype of the cells and delineating the rational of targeting these markers in liver cancer. In vitro experiments could further show that blocking of PD-1 increased IFN production and effectively enhanced the immune response. However, this effect was only present in PD-1^high^ HCCs [75]. 

Recently, single cell sequencing approaches became affordable and promising tools for translational science. These investigations are ideal to dissecting immune cell populations in the context of the diseased hepatic microenvironment as well as immunotherapies. A recent single cell sequencing analysis demonstrated a complex composition of highly diverse T cell subpopulations in HCC tumors [22]. A subgroup expressing high levels of exhaustion markers such as CTLA4 and PDCD1 was identified that stratified patients according to the clinical outcome [22]. Furthermore, complex composition of immune cells could be revealed and shown to be spatially different between intratumoral regions, extra-tumoral regions, ascites, and the peripheral blood [85]. While modulation of this immune cell contexture could be highly promising in a therapeutic setting, the clinical use of cellular compositions as predictors for therapy response needs to be evaluated.

Only one single cell study focused on the malignant cells in HCC so far. Analysis of the tumor and the TME identified VEGFA^high^ tumors that drive the TME reprogramming [86]. Consequently, further single cell analysis of T cells revealed different transcriptomic profiles in VEGFA^high^ tumors. These observations imply that a combination of vascular endothelial growth factor (VEGF) therapy and immunotherapy might help to overcome some non-response mechanisms.

Overall, results of these preclinical studies suggest that it is probably not enough to screen for widely expressed markers in the tissue and underline the importance of detailed characterization of the cellular compositions to shed light into cellular interactions to reveal context-dependent response mechanisms to immunotherapy.

For objective comparison of PD-L1 expression in clinical trials, mainly two different scoring systems have been established [87,88]. The tumor proportion score (TPS) calculates the percentage of PD-L1 tumor cells of all viable tumor cells, whereas the combined positive score (CPS) calculates the percentage of all PD-L1 positive cells (tumor cells, macrophages, lymphocytes) divided by all viable tumor cells [87,88]. PD-1/PD-L1 expression in tissue is associated with therapy response in melanoma, NSCLC, renal cancer and gastric cancer in large clinical trials [59,87,89,90,91].

Despite the promising results from the above-mentioned translational studies, explorative investigations performed on patients in clinical trials have failed to identify robust predictive markers that clearly identify patients likely to benefit from immunotherapy in HCC up to now. 

Clinical trials for HCC using ICI included both of the mentioned scores to predict response. The CHECKMATE-40 trial, investigating the anti-PD-1 antibody Nivolumab as a second line therapy in HCC reported response rates regardless of PD-L1 expression rates. PD-L1 expression was calculated using the TPS score (overall response rate (ORR) 26% in patients with PD-L1 expression >1% and ORR 19% of patients with PD-L1 expression <1%,). However, PD-L1 expression >1% could only be detected in 20% of the patient population. The lack of robust association indicates that PD-L1 expression on tumor cells cannot be used as a single binary marker for therapy decisions [40]. The phase II clinical trial KEYNOTE-224 used the anti-PD-1 antibody pembrolizumab after progression under sorafenib. Response to therapy was assessed using TPS as well as CPS score [41]. Only CPS score showed significant association with response to therapy. The proportion of CPS score positive patients in the KEYNOTE cohort has been reported as 42% [41]. Although the follow-up phase III study KEYNOTE-240 did not reach its clinical endpoint of OS, knowledge of PD-L1 expression and CPS score can be highly instrumental for future studies and are urgently awaited [70]. Noteworthily, different cutoffs and definitions about PD-L1 positivity have been used in clinical trials, which might have limited the comparability of the findings [92].

High tumor mutational burden (TMB), generally defined as over 10 mutations/mb, or microsatellite instability (MSI) are hypothesized to be intrinsically immunogenic [93]. Hence, TMB or MSI status were predictive for response to therapy with PD-1 checkpoint-inhibitors in several tumor entities [66,94,95,96]. However, compared to other tumor entities, HCC mainly has a low TMB of <10 mutations/Mb^low^ and MSI rates below 1% [75,96,97,98,99]. Given the low prevalence and only limited predictive ability of TMB, it emphasizes the need for more comprehensive molecular biomarkers [97]. 

Circulating immune cells and corresponding expression of checkpoint molecules have been intensively evaluated as predictive biomarker. Isolation and subsequent characterization would enable a closer and non-invasive therapy monitoring, which is not possible using tissue samples. However, only one study could identify an association of circulating immune cells and response to therapy so far. A higher expression of CD4^+^PD1^+^ cells in circulating peripheral blood mononuclear cells (PBMC) at baseline may predict a better response to tremelimumab treatment in HCC patients [100]. However, more recently, results from several clinical trials suggest that induction of a CD8 T cell response after CTLA-4 priming might enhance the anti-tumor efficacy of PD-1 inhibition [46]. This interesting observation should be pursued in future studies. 

Furthermore, high soluble PD-L1 levels are associated with a poor prognosis in HCC patients [101]. However, soluble PD-L1 could not be shown to be predictive under immunotherapy in HCC in contrast to other tumor entities [102,103].

Finally, studies have shown that the microbiome influences the immune system. Mice with liver tumors showed a better immune response and lower tumor burden when treated with antibiotics that reduced the overall bacterial burden in the gut but favor *Clostridium scindens*. Reduction of bacteria through antibiotics alters the composition of bile acids, which subsequently resulted in increased infiltration of NKT cells with anti-tumor function into the liver. On the other hand, gut microbiota has been shown to promote obesity-associated liver cancer by driving prostaglandin E2 (PGE2) production through higher expression of COX2. PGE2 eventually suppressed antitumor immunity and resulted in higher tumor burden of obesity-driven HCC [104]. Several studies have shown that the microbiome influences not only immune cells but also the efficiency of immunotherapy. Anti-PD1 therapy could be significantly improved by combing it with oral administration of *Bifidobacterium*, which resulted in reduced tumor growth of B16.SIY melanoma tumors [105]. Another study found the fecal transplantation of *Akkermansia muciniphia* can restore efficacy of anti-PD-1 immunotherapy, which was mediated by increasing the recruitment of CCR9^+^ CXCR3^+^ CD4^+^ T lymphocytes [105]. In human melanoma, anti-CTLA-4 therapy was associated with outgrowth of *Bacteroides fragilis*. Oral feeding of *Bacteroides fragilis* in germ-free mice resulted in restored therapeutic response to anti-CTLA-4 treatment [106]. Notably, a recent study focused on fecal samples from patients under immunotherapy as a predictive parameter and revealed a higher species richness in responding patients than in non-responders [107]. Furthermore, other studies suggest an association between commensal microbial composition and therapy response to immune therapy treatment in melanoma as well as HCC, whereas patient numbers were very limited (*N* = 8) [107,108]. Thus, data on the microbiome should be assessed as adjuvant information in future studies to identify its potential as a biomarker [109]. Data is mixed but it is clear that the composition of bacteria in the gut has influence and might predict response to immunotherapy and cannot be neglected. Sample acquisition in a hospitalized setting seems easy so that specifically response assessment and subsequent alteration of the treatment strategy based on the microbiome status seems to be reasonable.

Overall, while not yet conclusive in HCC, these findings provide the first mechanistic explanations of tumor cell biodiversity and why some patients may respond to therapy and others do not [86].

### 3.2. Molecular Subtyping of HCC

In the past, exome sequencing enabled a precise description of the mutational landscape in HCC including the identification of the most relevant oncogenic drivers (TERT, TP53, CTNNB1, AXIN1, ARID1A and ARID2) [18,110,111]. In 28% of all HCCs, potential targetable mutations were identified [112]. However, despite strong efforts, none of these potential biomarkers showed a significant survival benefit and could be implemented in clinical trials [18].

Analysis of the immune composition as well as the transcriptomic profile in HCC lead to the classification of inflamed “hot” tumors and non-inflamed “cold” tumors based on the presence of T cells, macrophages, B cells, PD1 signaling, and cytotoxic cytokines. Interestingly, “cold” tumors co-occur with WNT/CTNNB1 as well as chromosomal alterations of the tumor [18,111,113].

A retrospective analysis of genomic alterations of HCC patients undergoing immunotherapy revealed WNT1/CTNNB1 mutations to be associated with lower disease control rates (0% vs. 53%), shorter median progression free survival (PFS) (2.0 vs. 7.4 months), and shorter median OS [18,114]. This possible CTNNB1 immune excluded class could recently be confirmed in a translational mouse model [114,115]. Upregulation of β-catenin leads to an immune exclusion of the tumor and also resistance to anti-PD-1 therapy. These results conclusively illustrate, that other therapy modalities might be more suitable for cold or immune excluded and, potentially, other subclasses of HCC, and challenges the design of recent clinical investigations. In this context, molecular stratification of patients will become increasingly important and should be mandatory for future clinical trials.

## 4. Combination Treatments to Improve Therapy Response in HCC

### 4.1. Combination Therapies of Checkpoint Inhibitors

Given that the response to immunotherapy is restricted to 15–30% of the patients, the majority of the patients are not objectively responding or show a primary resistance to ICI. After initial studies on effectiveness of immunomodulatory drugs, new studies are focusing on mechanisms to increase therapy response [42]. The rationale behind combinations therapies is based on synergistic effects by CTLA4 induction followed by PD-1/PD-L1 blockade (Figure 2). Combination of different ICI blocks immune cell activation at different steps in their activation process. CTLA4 increases CD8 T cell activation in the priming phase in the lymph node as well as CD8 cell infiltration into the tumor. This enhances the effect of PD-1/PD-L1 blockade in the tumor microenvironment. The number of pretreatment or treatment induced intratumoral T cell infiltration correlates with clinical response to therapy, which emphasizes that the crucial factor for response to immunotherapy lies in releasing tumor-specific T cells [116].

The combination of checkpoint inhibitors antiPD-1/anti-PD-L1 plus anti-CTLA-4 antibodies have shown promising response rates of 40–60% in melanoma, NSCLC, and renal cancer [132,133,134]. Based on this, combination therapies of CTLA-4 and PD-1/PD-L1 blockade are currently under investigation [42].

In HCC, these combinations are also being actively pursed in clinical trials [45,135]. ORR rates for advanced non-resectable HCC in a phase II clinical trial (durvalumab (anti-PD-L1) and tremelimumab (anti-CTLA-4)) have been reported recently as 22% with 35% of the patients showing adverse events [136]. The phase III clinical trial (HIMALAYA) is currently underway [45]. However, the CHECKMATE-040 trial investigating nivolumab and ipilimumab could show overall response rates of 32% [42]. Further studies are required focusing on effectiveness versus increased adverse events. For a detailed list see Table 2.

### 4.2. Combination Therapies of Checkpoint Inhibitors and Anti-Angiogenesis

Another approach to enhance response to therapy explores additive effect of MKIs and ICI. It is well known that high VEGF levels in the TME modulate immunosuppressive T_regs_, macrophages and MDSCs, whereby promoting tumor growth [86]. Anti-angiogenic effects of MKIs mediated by VEGF inhibition can synergistically enhance the anti-tumor effects of ICI. Furthermore, Sorafenib effectively inhibits macrophage migration, macrophage induces epithelial-mesenchymal transition as well as macrophage-NK cell crosstalk in the liver [34,137]. In line with this, combination of pembrolizumab (anti PD-1) and Lenvatinib (MKI) reduced the secretion of immunosuppressive cytokines such as TGF-β and IL-10 and inhibited expression of PD-1 and Tim3, which enhanced antitumor immune response in a mouse model of hepatocarcinogenesis [138].

Further, the IMbrave150 phase III clinical trial confirmed the promising effects for the combination of atezolizumab plus bevacizumab, a direct VEGF inhibitor, in a first line treatment in HCC patients [48]. The experimental arm showed an ORR 33% versus 13% for sorafenib arm and median OS at 12 months was 67% versus 55%. These results have led to an FDA approval for the combination of bevacizumab and atezolizumab for advanced HCC and will likely become the new standard of care in advanced HCCs. Many other combination studies are currently underway (Table 2). Similar to the findings from the IMbrave150 study, ORR for pembrolizumab plus Lenvatinib have been reported 36% in a phase Ib clinical trial. Notably, 36% had serious treatment related adverse events [122]. Nevertheless, combination of ICI and MKIs show promising anti-tumor response rates. Many other studies are currently underway. For a detailed list see Table 2. 

### 4.3. Combination of Immunotherapy and Locoregional Therapy

A different approach to improve the response is to modulate the immunogenicity of tumors or to boost the immune system by combination of locoregional and/or radiotherapy with immunotherapy. This approach is based on releasing tumor antigens through cell death induced by locoregional therapy, which subsequently improves immunotherapy due to better antigen presentation. Thus, this combination is also discussed for neoadjuvant settings, when tumor burden is still high. In particular, antigen release and immunological response after irradiation has been extensively studied [139,140,141].

In 2004, de Broke et al. [142] could already show that RFA plus blocking CTLA-4 with tremelimumab causes a strong and durable antitumor response in a mouse model of B16 OVA melanoma cells. The same group showed that cryoablation and radiofrequency enables antigen loading of dendritic cells, which induced antitumor immunity [143], indicating that locoregional therapies could have more effects than just the local tumor elimination. The immunomodulatory effects caused by local therapies are of particular interest in the era of immunotherapies [144]. Different types of cell death can cause an immunogenic or non-immunogenic influence on the environment, whereas immunogenic cell death includes the release of calreticulin and other proteins of the endoplasmatic reticulum, which leads to activation of dendritic cells and improved tumor-antigen presentation for cytotoxic T cells [144]. A classical immunogenic cell death inducing chemotherapeutic is doxorubicin, which is most commonly used in TACE procedures in HCC patients [145]. MDSCs, which are increased in HCC patients, stimulate T_regs_ and correlate with HCC progression, [146,147] are decreased after RFA. However, patients with increased frequencies were more likely to recur after treatment. The effect of TACE or RFA on T cells seems to be stronger than surgery alone. After locoregional therapy, patients had a significant increase in GPC3 specific CTLs compared to patients undergoing surgery [140]. Radioembolization (Y90) on the other hand seems to have a sustained local as well as systemic immune response, that could be shown by an increase in TNFα in CD4, CD8 T cells, and APCs. The group could further demonstrate a prediction model based on peripheral blood samples before Y90 therapy [148].

However, response immunological response rates after locoregional therapy alone was not durable enough to prevent recurrence, underlining the potential of combination with immunotherapy [54]. The first combination therapy of tremelimumab and TACE, RFA, or cryoablation showed a good tolerability and an increase in intratumoral accumulation of CD8 T cells with good clinical response [43]. Remarkably, only lesions that were not directly treated were counted as tumor response, i.e., “abscopal effect” [43]. One combined clinical trial for HCC and CCA is investigating a combined immune checkpoint inhibition with ablative therapies (Durvalumab, Tremelimumab, TACE, RFA OR Cryoablation) (NCT02821754) [130]. For a detailed list of current clinical trials see Table 2.

While preclinical and early clinical data provides a clear rational for combination therapies, several open questions remain. In the context of combination therapy, the timing and sequence of corresponding therapies and identification of the best locoregional therapy in combination with the best immunotherapy are of particular interest. Further translational studies are also needed to improve the understanding of the exact molecular mechanisms involved in the response or failure of these combinations.

## 5. Translational Studies to Overcome Resistance to Immunotherapies

To detect molecular and cellular predictors of positive response to immunotherapy, animal models are widely employed in preclinical investigations, particularly syngeneic, genetically engineered, and humanized mice [149]. All of them harbor certain advantages and disadvantages, which should be carefully considered to accurately address the respective questions concerning immunotherapy.

### 5.1. Checkpoint Inhibitors

Investigation of immune checkpoint inhibitors using suitable models represents an important aspect of translational cancer research and is required for transitioning of crucial findings from bench to bedside. Detailed investigations on factors that are assisting immune evasion and contributing to the failure of classic chemotherapy are crucial [150]. Importance of CTLA-4 and PD-1/PD-L1 was thoroughly investigated in pre-clinical and early clinical models. Results revealed interesting and useful data for further translational implications and supported currently-used strategies in clinical trials [151,152].

Study of Brown et al. [153] tried to address mechanism of adaptive resistance to immunotherapy in the context of CTLA-4 checkpoint blockade. Results of this important study suggest that induction of Indoleamine 2,3-dioxygenase 1 (IDO1) typically appears in HCCs that are resistant to anti-CTLA-4 treatment, and that it is regulated in an IFN-γ dependent manner. These observations emphasized the importance of IDO1 as a regulator of adaptive resistance against anti-CTLA-4 treatment. Thus, combined therapy of IDO1 inhibitor and anti-CTLA-4 treatment emerges as a rational approach to improve the checkpoint-based treatments for the resistant types of HCC (Figure 2) [153]. In addition to increasing numbers of studies related to CTLA-4 therapy resistance, many new investigations aimed to delineate the fundamental mechanisms of PD-1/PD-L1-dependant immune tolerance in HCC [71,154]. In a chemically-induced HCC mouse model, exhaustion of tumor-antigen-specific CD8^+^ T cells, accumulation of PD-1 CD8^+^ T cells as well as T_regs_ was reported at the time of late tumor progression [71]. These findings encouraged authors to investigate a combination therapy of sunitinib and anti-PD-1 antibodies. This approach not only repressed adverse tumor features like immune evasion, but also directly reduced tumor burden and activated antitumor immunity [71].

To overcome immune tolerance, it is further important to explore more precise approaches to identify molecular components involved in immune evasion in HCC [155,156,157]. Polyinosinic-polycytidylic acid (polyIC), a double-stranded RNA, was firstly introduced as a molecule with potent liver tumor-inhibitory role only at the pre-cancer stage [155]. However, the potency of polyIC to treat advanced HCC was identified in a later study when it was combined with anti-PD-L1 antibody [156]. The mechanism of therapy response based on the ability of polyIC to enhance accumulation and activation of innate immune cells in the liver, particularly natural killer (NK) cells and macrophages, as well as to modulate adaptive immune functions by upregulation of PD-L1 in liver sinusoidal endothelial cells. These conditions sensitized the hepatic response to PD-L1 blockade and induced accumulation of active CD8^+^ T cells (Figure 2) [156]. These studies clearly imply that modulation of specific pathways can lead to sensitization of the tumors to PD-L1 blockade and improve the response in HCC mouse models. These interesting findings should be pursued in future pre-clinical and clinical trials.

Further efficacy improvements of checkpoint inhibitors could be achieved through disruption of pathways involved in epigenetic regulation. For example, combination of histone deacetylase inhibitor belinostat with anti-CTLA-4, or combination of anti-CTLA-4 plus anti-PD-1 antibodies could lead to complete tumor rejection in a mouse HCC model [158]. Moreover, another study suggests that PD-L1 blockade and SIRT7 inhibition could be a more efficient clinical option to target HCC (Figure 2) [159]. Overall, these results provide a rationale for testing epigenetic modulators in combination with checkpoint inhibitors to enhance their therapeutic activity in patients with HCC.

All together, these animal studies clearly demonstrate the importance of the cellular composition and balance of pro- and anti-tumor immune cells for effectiveness of immunotherapy. Results clearly delineate capacity of epigenetic regulators to improve the immunotherapy response. 

### 5.2. Application of Neoantigens and Oncolytic Viruses in Immunotherapy

One of the strategies to induce a positive immune response against cancer is the activation of CD8^+^ T cells, either by antigen-presenting or by tumor cells. In this context, particularly interesting are the neoantigens that arise as a result of tumor-specific mutations, which could be effectively used for development of novel therapeutic approaches [52]. An effective way to increase neoantigen presentation to CD8^+^ T cells in the tumor/-microenvironment is induction of cellular death by using various approaches, such as local ablation therapy or oncolytic viruses (OV) [53,160].

In a recent study, release of neoantigens was induced in an orthotopic mouse HCC model by applying image-guided stereotactic radiation. The treatment generated insufficient CD8^+^ T cell mediated immune response due to feedback inhibition of T cells by increased PD-L1 expression on macrophages. Interestingly, antitumor effect was enhanced when combining stereotactic radiation with anti-PD-1 treatment. This approach promoted adaptive immunity and infiltration of CD8^+^ cytotoxic T cells in the tumor, but only in a transient manner [72]. 

Great potential of OVs for the cancer treatment has been recognized in preclinical animal models as well as in human cancer patients [161,162]. Particularly interesting is application of oncolytic viruses in immunotherapies, which are specifically designed to selectively lyse cancer cells and to induce specific anti-tumor immunity. However, despite of a number of OVs that were examined in the preclinical studies, a low number entered into the clinical trials [161,162]. The most advanced of them is JX-594 (Pexa-Vec), which has entered a phase 3 randomized clinical study (PHOCUS). In this trial, the main objective is to determine if treatment with JX-594 and sorafenib increases survival in patients with advanced HCC who did not previously receive systemic therapy (NCT02562755). Therefore, development of new preclinical models to evaluate the effects of oncolytic viruses in HCC will pave the road for advanced clinical trials and speed up development of new cancer treatments. In line with that, new generations of OVs have been developed with greater potential to specifically target tumor cells and stimulate the immune response [163,164]. Recently, Nakatake et al. [163] examined the antitumor activities and immune response of third-generation HSV T-01 in HCC cell lines and mouse xenograft models. Application of the virus successfully led to increased expression of MHC class I molecules on tumor cells, which further stimulated CD8^+^ T cell-mediated immune response. Importantly, viral treatment induced only antitumor effects without affecting normal cells, demonstrating great potential and specificity of this approach [163]. The capability of HSV-1 was further examined in a study where a novel HSV-1 vector, Ld0-GFP, was developed. Administration of the vector clearly showed increased tumor selectivity and oncolytic capacity against HCC by enhancing cell apoptosis in different mouse models. Overall, viral-induced oncolysis provoked strong immunogenic cell death by activating the immunogenic cell death pathway [165]. Despite the above mentioned OVs, several other viruses have also been explored in the context of HCC. 

Overall, both exploration of neoantigens and direct tumor lysis by OV, show great translational value, as some of the investigated models and are currently investigated in clinical trials.

### 5.3. Targeting HCC Biomarkers–Vaccines, Antibodies, and Cytokines

Targeting a specific marker or a component of immune defense in HCC could be an effective way to overcome resistance commonly observed with classic chemotherapies [111]. New opportunities are emerging as specialized anti-cancer vaccines are developed and tested in animal models [166]. Most compelling are the vaccines that specifically target HCC-associated markers such as AFP and GPC3 (approach known as “antigen-defined”) [167,168,169]. Many studies exploited the potential of AFP for designing an effective HCC vaccine [170,171,172,173]. In order to induce immune response, different approaches such as application of AFP plasmid DNA, dendritic cell (DC) transduction with viral vectors, or a combination of AFP with heat shock proteins have been evaluated [170,171,172,173]. However, the most promising results of AFP cancer immunization were achieved through production of epitope-optimized AFP, which effectively activated CD8^+^ T cells and generated potent antitumor effects in HCC mouse model [174]. Several studies tried to target the activation of GPC3, a glycoprotein overexpressed in many HCC tissues, in order to design an effective vaccine [169]. Preclinical evidence suggests that intravenous injection of the GPC3-coupled lymphocytes can induce a strong anti-HCC effect by regulating systemic and local immune responses [169].

In addition to the above-mentioned vaccine-based approaches, a growing number of antibodies are produced to eradicate or neutralize specific molecular or cellular targets [61]. Several antibodies were also successfully targeted including GPC3, a member of the TNF receptor family CD137, transmembrane four L6 family member 5 protein, and fibrinogen-like protein 1 [175,176,177]. These investigations also demonstrated various degrees of anti-tumor and immune-modulatory capacity. In addition, immune modulation directed against liver cancer can be initiated by a release of cytokines involved in cellular antitumor response [178,179]. For instance, IL-33 release in murine HCC showed to markedly inhibit tumor growth via activated CD4^+^ and CD8^+^ T cells, in IL-33-expressing tumor-bearing mice, while IL-18/IL-12 cytokine therapy was effective in tumor regression prompted by induction of NK cells [178,179,180]. 

Taken together, development of different strategies to target specific HCC biomarkers and to modulate cytokine release shows big potential in immunotherapy of HCC.

### 5.4. Adoptive Cell Transfer and CAR T Cells

The basic principle of adoptive cell transfer is to disrupt the immune tolerance of tumors and, consequently, to suppress the growth and survival of tumor cells. This is achieved when lymphocytes are extracted from the patients, with the purpose of modification and amplification in vitro, and, subsequently, transferred back into the patient. This method enhances the overall specific antitumor effect [181]. Most of the recent studies on adoptive cell transfer were focused on targeting GPC3 [182]. In a seminal study, GPC3-specific CD8^+^ T cells were engineered and subsequent antitumor capabilities in HCC xenograft mice were tested. This approach showed only partial response, as CD8^+^ T cells were only able to slow down tumor growth in whole-body irradiated mouse model. Further, immunodeficient model displayed higher suppression of tumor growth. In this model, failure of significant tumor response was consequence of a lack of CD8^+^ T cell infiltration into the tumor and by mosaic-pattern of GPC3 expression which could be enhanced in future studies [182]. 

However, more recently, CAR T cell-based therapy gained increasing attention as a potentially more efficient method to target tumor cells [183,184,185,186]. Earlier studies have already proven the potential of CAR T cells to effectively target GPC3^+^ HCC cells in vivo. Anti-GPC3 CAR T cells successfully suppressed tumorigenesis in subcutaneous tumors and significantly affected tumor growth in subcutaneous and orthotopic xenografts [183]. Similar observation was noted in a patient-derived xenograft model. CAR T cells directed against GPC3 eradicated tumors from patient derived xenografts that showed less aggressive phenotype and lacked PD-L1 expression, while on the contrary, GPC3 CAR T cells were less potent in aggressive tumors with high PD-L1 expression. This all emphasized the potential of combination therapy with immune checkpoint inhibitors [185]. Except of combining GPC3-CAR T cells with checkpoint inhibitors, Wu et al. investigated potential application of sorafenib to induce additive effects. The authors reported that sorafenib enhanced the antitumor effects of CAR T cells, partially by promoting IL12 secretion by TAMs as well as promotion of apoptosis in immunocompetent and immunodeficient mouse models of HCC [186]. It is also important to mention that NK cells were investigated in the context of chimeric antigen receptor with promising results. This makes NK cell-based therapy as a novel treatment option for patients with GPC3^+^ HCC [184]. 

Major studies on CAR T cells in HCC have been conducted with the main focus on GPC3. They shed more light on this complex topic and provided evidence for further investigations to define new targets for CAR T treatments. However, heterogeneous intra- and inter-tumoral expression of surface antigens as targets for CAR T-based approaches including GPC3 severely complicate this approach in human HCC.

### 5.5. Targeting Cross-Communication between MDSCs and the TME

The chronically altered tumor microenvironment in HCC, particularly liver fibrosis, significantly shapes and modulates the course of HCC development specifically by reprogramming an immunosuppressive mechanism [187]. Accumulation of monocytic MDSCs (M-MDSC) in fibrotic tumor microenvironment in orthotopic mouse model can significantly reduce the number of TILs and increase tumorigenicity [187]. Recent investigations have revealed that contribution to immune tolerance and higher tumorigenicity was closely connected to the interaction between HSC from the fibrotic microenvironment and M-MDSC [187]. Namely, HSC could induce M-MDSC accumulation and immunosuppression through p38 MAPK-mediated enhancer reprogramming. Treatment with BET bromodomain inhibitor significantly reduced the level of M-MDSC and increased the level of tumor-infiltrating CD8^+^ T cell. When BET bromodomain inhibitor treatment was combined with anti-PD-L1 therapy, synergistic effects of the treatments led to tumor eradication and prolonged survival in this fibrotic-HCC mouse model. Therefore, targeting cross-communication (HSC-M-MDSC) in fibrotic liver could be a novel therapeutic strategy that could improve the efficacy of anti-PD-L1 therapy [187]. More evidence on how the response to PD-1/PD-L1 therapy could be further improved is presented by indirect modulation of IL-6 signaling, a major immune-modulatory cytokine in the liver [188]. Inhibition of *Ccrk* and CCRK/EZH2/NF-κB/IL-6 signaling cascade can bypass MDSC-mediated IFN-γ^+^ TNF-α^+^CD8^+^ T cell exhaustion and cause reduction in tumorigenicity. More importantly, inactivation of this signaling cascade paralleled with administration of anti-PD-L1 therapy could improve efficacy of checkpoint inhibitors in orthotopic HCC model and prevent immune evasion [188]. 

### 5.6. Targeting MDSC, TAMs, and Innate Immunity Interaction for HCC Prevention

Given the fact that macrophages promote HCC progression, therapeutic manipulation of this interaction is of major interest. This includes the inhibition of monocyte recruitment into the liver, polarization from M1 to M2 macrophages, inhibition of TAM associated cytokines, or direct inhibition of macrophages present in the tumor [189,190,191,192]. Blocking of CCL2-CCR2, which inhibits monocyte recruitment, was revealed to be effective in HCC mouse models. Namely, this approach increased tumor infiltrating macrophage numbers, promoted polarization into a M2 phenotype as well as enhanced a T cell antitumor response [193,194]. Moreover, treatment with Mi-RNA-26a effectively suppressed tumor growth by downregulating colony stimulating factor-1 (CSF1 or M CSF), which further inhibited macrophage recruitment [195]. Blocking of CSF1 and CSF1 receptor (CSF1R) has also been demonstrated to enhance the effectiveness of immune checkpoint inhibitors [157]. A recent study has reported that Osteopontin facilitates chemotactic migration and M2-like polarization of macrophages and promotes the expression of PD-L1 in HCC. These events are mediated via activation of CSF1-CSF1R pathway in macrophages, which leads to increase of immunosuppressive cytokine levels. Therefore, blocking the CSF1/CSF1R pathway could effectively prevent macrophage recruitment and M2 phenotype polarization, activate CD8^+^ T cells, and sensitize HCC to anti-PD-L1 immune checkpoint blockade (Figure 2) [157]. PLX3397 also inhibits CSF-1R and could prevent tumor growth in a murine HCC model by macrophage reprogramming [192]. Another agent, baicalin (a flanonoid), repolarized macrophages into M1-like macrophages in an orthotopic mouse model of liver cancer [196]. All these translational findings suggest potential combination therapies to reprogram the immunological TME. 

From the perspective of innate immunity, NK cells are considered to be one of the key players in the prevention of HCC [29,197]. They exert a critical role in the antitumor immunity by modulating both, innate immunity as well as activation of adaptive immunity, by cross-talking with DCs and promoting a T helper cell (Th)1-mediated immunity [29]. However, positive role of NK cells in fight against cancer has often been impaired in HCC [198,199]. It was already shown that MDSCs in patients with HCC suppress the innate immune system by diminishing autologous NK cell cytotoxicity and cytokine secretion. These events activate immune suppressor network and allow the tumors to evade the host immune response [200]. Earlier studies in mice determined that inhibition of NK cell cytotoxicity is contact-dependent, where MDSCs inhibit IL-2-mediated NK cell activation, by dysregulating Stat5 signaling [201]. More evidence on the dysregulation of NK cells by MDSCs was obtained in the liver cancer-bearing mouse model. Results showed that increased levels of MDSC directly influenced NK cell function by inhibition of their cytotoxicity and IFN-γ production. The main mediator of NK cell suppression was membrane-bound TGF-β1 on MDSC [29]. Taken together, disruption of interaction between MDSC and components of innate immunity, particularly NK cells, represents an attractive approach to confront development of HCC.

## 6. Conclusions and Future Direction

Primary liver cancer develops in a fine-tuned and very complex microenvironment. Immune cell composition and interactions with tumor as well as stromal cells play a crucial role in development and progression of liver cancer. Modern immune-oncological approaches in HCC significantly expanded the landscape of active compounds in HCC over the recent years. However, efficacy of targeting individual aspects of immune response, including checkpoint molecules, remain decisively low. Furthermore, predictive biomarkers for therapy response are still largely missing. Thus, implementation of results and different approaches from preclinical, translational studies might be of utmost importance to identify novel cellular or molecular targets that synergistically could improve currently used strategies. Herein, an improved understanding of the landscape of immune-oncological alterations and rationale for subsequent molecularly-guided combination therapies are urgently needed. Up till now, our current understanding remains incomplete and precise dissection of intra- and inter-tumoral heterogeneity using single cell sequencing approaches still is in its infancy for HCC. In addition, detailed knowledge on the immune-cell contexture will add additional layers of complexity that requires detailed preclinical models that closely resemble authentic human HCC. However, a better understanding of molecular interaction and pathways on a cellular level is imperative to develop new treatment regimens or combination of regimes. As the knowledge on molecular and immune-modulatory pathways evolve, the corresponding context of application and genetic background will need to be tightly controlled to ultimately implement the translational finding, overcome therapy resistance, and increase clinical response rates. Nevertheless, recent findings form clinical trials on different combination therapies are highly promising and will likely further shape the therapeutic landscape and enter the clinical practice of HCC treatment.

## Figures and Tables

**Table 1:** Currently approved immunotherapy in hepatocellular carcinoma (HCC)

Target Molecule	Drug Name	Company
PD-1	Nivolumab	Bristol Meyer Squibb
PD-1	Pembrolizumab	Merck
PD-L1	Atezolizumab (in combination with bevacizumab)	Roche
CTLA-4	Ipilimumab	Bristol Meyer Squibb/Medarex

Abbreviations: PD-1 (programmed cell death protein 1), PD-L1 (programmed cell death ligand 1), CTLA-4 (cytotoxic T-lymphcyte-associatet protein 4).

**Table 2:** Summary of clinical trials for immunotherapy (mono- and combination therapies/completed and ongoing) in HCC.

Author (Year)	Phase/Trial Name	Target	Therapy Regimen	ORR	PFS (Months)	pts	DCR	Additional Information	Status
Monotherapy									
Sangro (2013) [44]	II	CTLA-4	Tremilimumab	17.6% (3 PR)	6.48	21	76.4%		completed
El-Khoueiry (2017) [40]	II CHECKMATE-40 Second line	PD-1	Nivolumab	20% (3 CR, 39 PR)	4.0	214	64% (37% over 6 months)	PD-1^high^ ORR 26%, PD-1^low^ ORR 19%, 9 months OS 74%, KM median not reached yet	completed
Zhu (2018) [41]	II KEYNOTE-224 Second line	PD-1	Pembrolizumab	17% (1 CR, 17 PR)	NR	104	64%	Positive correlation of ORR and TPS score	completed
Finn (2020) [117]	III KEYNOTE-240 Second line	PD-1	Pembrolizumab	18.3% (6 CR, 45 PR)	3.0	413	62.2%, (31% over 6 months)	OS 13.9 months	negative trial
Yau (2019) [73]	III CHECKMATE459 First line	PD-1	Nivolumab vs. Sorafenib	15% (14 CR, 43 PR)	3.7	743	-	PD-L1^high^ ORR 28%, PD-L1^low^ ORR 12%	negative trial
Qin (2019) [118]	III Rationale 301 First line	PD-L1/PD-L2	Tislelizumab vs. Sorafenib	-	-	-	-		ongoing
Exposito (2018) [119]	III CHECKMATE-9DX Adjuvant	PD-1	Nivolumab	-	-	530	-		ongoing
Combination of immunotherapies									
Yau (2019) [42]	I/II CHECKMATE-40	PD-1 + CTLA-4	Nivolumab + Ipilimumab	32% (4 CR, 12 PR)	-	148	54%	12 months OS 61% PD-1^high^ and PD-1^low^: no difference	ongoing
Kelley (2020) [46]	I/II	PD-L1, CTLA-4	Durvalumab + Tremelimumab	22%	-	75		Median OS 18.7 months	ongoing
Abou-Alfa (2018) [45]	III HIMALAYA	PD-L1, CTLA-4	Durvalumab + Tremelimumab vs. Durvalumab vs. Sorafenib	-	-	-	-		ongoing
Kaseb (2019) [120]	II Neoadjuvant + adjuvant	PD-1, CTLA-4	Nivolumab + Ipilimumab	37.5% (3 CR)	-	8	-		ongoing
Combination with MKI									
Bang (2019) [121]	Ib	PD-L1 + VEGF	Durvalumab + Ramucirumab	11% (3 CR+PR)	4.4	28	61%	PD-L1^high^ ORR 18%, DCR 73%	ongoing
Zhu (2020) [122]	Ib KEYNOTE 524 First line	PD-1 + MKI	Pembrolizumab + Lenvatinib	36% (1 CR, 35 PR)	8.6	30	60%		ongoing
Llovet (2019) [123]	III LEAP002 First line	PD-1 + MKI	Lenvatinib + Pembrolizumab vs. Lenvatinib	-	-	750	-		ongoing
Xu (2019) [124]	I Second line	PD-1 + MKI	Camrelizumab + Apatinib	50% (8 PR)	5.8	39 (16 HCC)	93.8%	OS NR	ongoing
	II IMMUNIB First line	PD-1 + MKI	Nivolumab + Lenvatinib	-	-	est. 50	-		ongoing
Pishvaian (2018) [125]	Ib	PD-L1 + VEGF	Atezolizumab + Bevacizumab	34% (1 CR, 22 PR)	14.9	68	78% (50% over 6 months)		ongoing
Finn (2018) [48]	III IMbrave150 First line	PD-L1 + VEGF	Atezolizumab + Bevacizumab vs. Sorafenib	33% (33 CR, 75 PR)	6.8	325	72.3%		ongoing
Yau (2020) [47]	II CHECKMATE 40	PD-1 + CTLA-4 + MKI	Nivolumab + Cabozantinib vs. Nivolumab + Ipilimumab + Cabozantinib	26% (9 PR)	6.8	71	83%	71% grade III-IV AEs, discontinuation in 20%	ongoing
Kudo (2019) [126]	Ib VEGF Liver 100 First line	PD-L1 + MKI	Avelumab + Axitinib	13.6%	5.5	22	68.2%	OS 12.7 months	ongoing
Kelley (2019) [127]	III COSMIC-312 First line	PD-L1 + MKI	Atezolizumab + Cabozantinib vs. Sorafenib	-	-	640	-		ongoing
Knox (2019) [128]	III EMERALD 2 Adjuvant	PD-L1 + VEGF	Durvalumab + Bevacizumab	-	-	-	-		ongoing
Combination with locoregional therapy									
Duffy (2017) [43]	I/II	CTLA-4 + locoregional	Tremilimumab + TACE/RFA	26.3%	7.4	32	-	OS 12.3 months	completed
Sangro (2020) [129]	III EMERALD 1 adjuvant	PD-L1 + VEGF + locoregional	Durvalumab + Bevacizumab + TACE	-	-	600	-		ongoing
Charalampos (2019) [130]	II adjuvant	PD-L1 + CTLA-4 + locoregional	Durvalumab + Tremilimumab + TACE/RFA/cryoablation	20% (2 PR)	7.8	22 (10 HCC)	60%	OS 15.9 months	ongoing
	II IMMULAB	PD-1 + locoregional	Pembrolizumab + RFA/MWA	-	-	-	-		ongoing
	II PLTHCC	PD-1 + MKI+ locoregional	Immunotherapy + Lenvatinib + TCAE	-	-	-	-		ongoing
Popovic (2019) [131]	Ib CaboNivo Neoadjuvant in locally advanced HCC	PD-1 + MKI + resection	Nivolumab + Cabozantinib	-	-	15	-		ongoing

## Abbreviations

Ab	Antibody
AFP	alpha-fetoprotein
APC	antigen presenting cells
CCRK	cell cycle-related kinase
CPS	combined positive score
CSF1	colony stimulating factor 1
CSF1R	colony stimulating factor receptor 1
CTLA-4	cytotoxic T lymphocyte antigen 4
CXCR-4	CXC receptor type 4
DC	dendritic cell
GPC3	glypican-3
HCC	hepatocellular carcinoma
HSC	hepatic stellate cell
ICI	immune checkpoint inhibitor
IDO1	indoleamine 2,3-dioxygenase 1
LAG3	lymphocyte activation gene 3
M-MDSC	monocytic MDSC
MDSC	myeloid-derived suppressor cells
NAFLD	non-alcoholic fatty liver disease
NASH	non-alcoholic steato hepatitis
NF-κB	nuclear factor-κB
NK cells	natural killer cells
NSCLC	non-small cell lung cancer
OR	overall response rate
OS	overall survival
PBMC	peripheral blood mononuclear cells
PD-1	programmed death protein 1
PD-L1	programmed death ligand 1
PFS	progression free survival
PSC	primary sclerosing cholangitis
TAM	tumor-associated macrophages
Th	T helper
TIL	tumor infiltrating lymphocytes
TIM3	T cell membrane protein 3
TKI	tyrosine kinase inhibitor
TME	tumor microenvironment
TPS	tumor proportion score
T_reg_	regulatory T cells
VEGF	vascular endothelial growth factor
